# A call for improving lithium literacy among clinicians and patients

**DOI:** 10.1186/s40345-022-00250-y

**Published:** 2022-03-01

**Authors:** Fabiano A. Gomes, Elisa Brietzke, Michael Bauer, Robert M. Post

**Affiliations:** 1 Department of Psychiatry, Queen’s University, Kingston, ON Canada; 2 Department of Psychiatry and Psychotherapy, Technische Universität Dresden, Dresden, Germany; 3 George Washington University School of Medicine, Washington, DC USA

Despite overwhelming evidence that lithium prevents episodes of bipolar disorder and suicide attempts and suicide, (Tondo et al. 2019) there has been a consistent decline in its use in the past decades, especially in North America. It was with great pleasure that we read two recent articles addressing lithium prescriptive patterns in the Journal. Sköld et al. (2021) presents an interesting study investigating regional differences in lithium prescription in Sweden and showed that higher frequency of lithium use was associated with lower rates of recurrences. Even if considered the already high rates of lithium use, the authors conclude that there would be more benefit if the clinical use of lithium was increased. In the same vein, Pérez de Mendiola et al. (2021) reported their findings of a nationwide survey among Spanish psychiatrist regarding lithium use. They concluded that lithium use in Spain is in line with the international guidelines and that the first reason not to prescribe lithium was related to adverse effects and not its practical use or effectiveness. These results, although not new, illustrate that there is still a lot of educational and advocacy work to be done in order to address the underuse of lithium in clinical practice.

There are several factors are related to this phenomenon and include the introduction and marketing of new agents, usually perceived as more tolerable, and the emphasis on side-effects of lithium. When balancing the clinical benefits with the fear of short and long-term complications, clinicians and patients may choose a “cautionary’’ approach, losing the opportunity to use a potentially disease-modifying medication (McIntyre et al. 2020). A key component of increasing lithium use is directly providing information to both clinicians and patients about the multiple assets of lithium beyond its ability to treat and prevent mania and depression. It increases neurogenesis, hippocampal and cortical volume; ameliorates white matter tract abnormalities; may prevent cognitive decline; has neuroprotective effects against multiple neuropsychiatric illnesses (Puglisi-Allegra et al. 2021); has anti-suicide effects in persons with mood disorder and the general population (Matto et al. 2020), and increases the length of telomeres by a direct effect on the enzyme telomerase (Squassina et al. 2017). Since stress and episodes of depression shorten telomere length and increase the risk of multiple illnesses, lithium’s reparative effect on telomere length could have many health benefits.

Many patients have heard only about the negatives of lithium, such as weight gain, kidney and thyroid dysfunction, tremors, and increased urination, or the hypothetical that it decreases creativity. Each of these can be countered with specific information on how to minimize or manage the potential side-effects (Gitlin 2016). Clinicians must be educated and actively inform their patients about the over-emphasized negatives, as well as the positives of lithium which are usually not known. This focus on dealing with patient’s misconceptions and educating them about lithium’s multiple positives will have two benefits. It will teach the practitioner the data for themselves and focus on the most important target of lithium use—the patient.

Learning how to covey this information to the patient should be an important part of educational interventions aimed to increase the use of lithium and should target clinicians early in their training (Tondo et al. 2019). Active learning methods, including hands-on workshops, games and role-playing may help prescribers to deal with patients that reject the use of lithium. Telling patients about increasing their hippocampal volume and the length of their telomeres may even have greater impact than the data that lithium prevents mood episodes.

## Data Availability

Not applicable.
